# Аtherosclerosis‐like changes in the rabbit aortic wall induced by immunization with native high‐density lipoproteins

**DOI:** 10.1002/iid3.339

**Published:** 2020-08-13

**Authors:** Kseniya Fomina, Liubov Beduleva, Igor Menshikov, Abdulkadhim Zerjawi, Alexey Terentiev, Alexandr Sidorov, Tatyana Khramova, Nadezhda Abisheva, Anna Gorbushina

**Affiliations:** ^1^ Laboratory of Molecular and Cell Immunology, Department of Immunology and Cell Biology Udmurt State University Izhevsk Russian Federation; ^2^ Laboratory of Biocompatible Materials Udmurt Federal Research Center UB RAS Izhevsk Russian Federation

**Keywords:** antibodies specific to HDL, aortic wall metaplasia, atherosclerosis, cholesterol, chondrocyte‐like cells

## Abstract

**Introduction:**

A high level of total cholesterol or low‐density lipoprotein (LDL) cholesterol is considered the main cause of atherosclerosis and cardiovascular disease. For this reason, experimental atherosclerosis is induced by creating high blood cholesterol in animals. However, the hypothesis that atherosclerotic processes are mostly caused by immune (autoimmune) mechanisms has recently been gaining traction. At the same time, no experimental model has been developed that clearly demonstrates the autoimmune mechanism by which atherosclerosis develops and reproduces the full picture of atherosclerosis solely by means of an immune response, without resorting to additional interventions such as a high‐cholesterol diet or the use of genetic models of hyperlipidemia. Previously, we were able to induce atherosclerosis‐like lesions in the aorta and the development of pericardial fat in rats by immunizing them with human native lipoproteins. The purpose of this study was to test whether atherosclerosis can be induced in normocholesterolaemic rabbits by immunizing them with human native high‐density lipoproteins (hnHDL).

**Methods:**

Rabbits were immunized with hnHDL. Aortic wall structure, plasma cholesterol level, and antibodies against HDL were studied.

**Results:**

Immunization with hnHDL was found to cause atherosclerosis‐like lesions in the rabbit aorta such as adipocytic and chondrocytic metaplasia, proteoglycan deposits, leukocytic infiltration. Atherosclerosis‐like lesions developed in the aorta of hnHDL‐immunized rabbits against a background of normal blood LDL‐cholesterol level. Therefore, a high plasma cholesterol level is not the sole cause of atherosclerosis. The immune response against HDL is an independent cause of atherogenesis.

**Conclusions:**

A rabbit model of atherosclerosis caused by immunization with hnHDL can be widely used to examine the mechanisms occurring during atherogenesis.

## Graphical abstract

Immunization with human native high‐density lipoproteins (hnHDLs) induces atherosclerosis‐like lesions in the rabbit aorta such as adipocytic and chondrocytic metaplasia, proteoglycan deposits, and leukocytic infiltration. Atherosclerosis‐like lesions develop in the aorta of hnHDL‐immunized rabbits against a background of normal blood low‐density lipoprotein‐cholesterol level. Consequently, the immune response against HDL may be an independent cause of atherogenesis, and HDL is the potential target of the immune attack that leads to atherosclerosis.

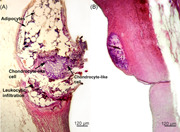

## 1 INTRODUCTION

Experimental modeling of atherosclerosis traditionally starts from the idea that atherogenesis is caused by an abnormality of lipid metabolism, namely low‐density lipoprotein (LDL)‐hypercholesterolemia. The general scientific consensus is that the excess plasma LDL leads to the accumulation and oxidation of lipoproteins in the vessel wall, and this in turn induces arterial inflammation, the advanced stage of which is an atherosclerotic plaque.^1^, ^2^ For this reason, experimental atherosclerosis is induced by creating high blood cholesterol in animals. This is achieved either with a high‐cholesterol diet^1^ or by using animals that develop hypercholesterolemia as a result of genetic defects, such as the deletion of apolipoprotein E (Apoe−/−) or the destruction of LDL receptors (Ldlr−/−).^2^ However, the understanding of the mechanism by which atherosclerosis develops is currently shifting.^3^ There is increasing support for the hypothesis that a primary role in initiating atherosclerosis is played by immune responses, particularly autoimmune responses.^3^, ^4^ Over the lengthy period during which the autoimmune hypothesis of atherosclerosis has been developed and strengthened, the role of the immune response against oxidized LDL, the heat shock proteins (hsps) of microorganisms, apolipoprotein A‐1 (main protein constituent of high‐density lipoprotein [HDL]) and vessel wall antigens have been studied.^5^, ^6^, ^7^ The experimental data on the role of antibodies against oxidized LDL are contradictory.^5^, ^7^, ^8^ In our own studies, we were unable to confirm the role of antibodies against oxidized LDL in the development of atherosclerosis in rats.^8^ Wick et al^5^ induced changes in the vessel wall of rabbits by immunizing with hsp‐60/65. At the same time, they showed that antibodies to hsps alone are not sufficient to produce irreversible changes in the vessel wall and plaque formation. For this, hsp‐65 immunization must be combined with dietary cholesterol overload.^5^ Immunization with vessel wall antigens obtained from plaques was also unsuccessful.^5^ Thus, no experimental model has been obtained that would clearly demonstrate the immune mechanism of atherosclerosis development and would reproduce the full picture of atherosclerosis solely by means of an immune response, without resorting to additional interventions such as a high‐cholesterol diet or the use of genetic models. Furthermore, the questions of which antigens are the targets of the immune attack that leads to atherosclerosis and how these immune responses result in plaque formation remain unanswered.

Previously, we were able to induce changes in the aortic wall similar to those observed in the early stages of human atherosclerosis, and also to produce visceral obesity in normocholesterolaemic Wistar rats by a single immunization with human native HDL (hnHDL) or hnLDL.^8^ The reason we chose native and not oxidized human lipoproteins as the antigenic inducer of atherosclerosis was the known principles by which other autoimmune diseases—specifically collagen‐induced arthritis in rats—have been successfully induced, namely by immunization with a target antigen in native form and the use of heterologous antigen.^9^ Rats immunized with hnHDL or hnLDL that as a result produce antibodies against native lipoproteins were found to have pericardial fat, increased visceral adipose tissue volume, inflammation in the aortic wall as identified by the accumulation of leukocytes therein, and destruction of the intima and disruption of the media structure.^8^ The drawback of this rat experimental model of atherosclerosis and visceral obesity is the absence of the true atherosclerotic plaques that are typical of human atherosclerosis. The reason atherosclerotic plaques cannot form in rats may be the particular features of aortic wall morphology in rats. Therefore, to obtain a true autoimmune model of atherosclerosis and confirm the role of immune responses to native lipoproteins in the development of atherosclerosis, we attempted to induce atherosclerosis in rabbits by the same method as in rats, namely by immunizing with native HDL.

## 2 MATERIALS AND METHODS

### 2.1 Rabbits

White giant male rabbits (n = 11) were obtained from the Udmurt Veterinary Diagnostic Center (Izhevsk, Russia). Before and during the experiment, the animals were maintained on standard rabbit chow without supplements and had unrestricted access to water.

### 2.2 Ethics statement

Animal experiments were performed in accordance with the ARRIVE guidelines, the U.K. Animals (Scientific Procedures) Act, 1986, and EU Directive 2010/63/EU for animal experiments. The protocol and procedures employed were ethically reviewed, and approved by the Bioethics Committee of Udmurt State University (date: 15 February 2017/No. 1701).

### 2.3 Immunization

At 2 months of age, the rabbits (n = 4) were immunized with human native HDL (Kalen Biomedical). The lipoproteins were administered as a single intradermal injection of 200 ug (by protein) per rabbit in incomplete Freund's adjuvant (IFA) (Sigma‐Aldrich). Control rabbits received a subcutaneous injection of IFA (n = 4) or 0.15M NaCl (n = 3).

### 2.4 Tissue preparation and histology

The hnHDL‐immunized rabbits and the control animals were dissected 39 to 47 weeks after immunization, IFA injection or 0.15M NaCl injection, respectively. The rabbits were killed by an overdose of intravenous pentobarbital sodium and perfused with phosphate‐buffered saline (PBS) followed by Immunofix (Bio Optica Milano Spa, Italia) through the left ventricle. Aortic specimens were fixed for 24 hours in immunofix and embedded in paraffin for light microscopy. Cross‐sections, 5‐μm thick, were stained with hematoxylin and eosin to visualize morphology as well as by the pentachrome method proposed by K. Doello for staining the extracellular matrix.^10^

### 2.5 Determining plasma LDL and HDL‐cholesterol levels in the rabbits

Blood samples were taken from an ear vein of each hnHDL‐immunized rabbit or IFA‐injected animal at 2, 4, 6, and 8 weeks post‐HDL immunization. LDL cholesterol and HDL cholesterol were measured by direct homogenous assay using LDL cholesterol liquicolor No. 10094 and HDL cholesterol liquicolor No. 10084 Kits, respectively (HUMAN Gesellschaft für Biochemica und Diagnostica mbH, Wiesbaden, Germany). Spectrophotometer Genesys 10S UV‐Vis, Thermo Fisher Scientific Inc, (Сenters for the collective use of scientific equipment, UdSU) was used.

### 2.6 Measuring plasma lipid composition in the rabbits

In addition, the ratio of serum lipid fractions was studied in each hnHDL‐immunized rabbit or IFA‐injected animal at 4 and 8 weeks after immunization. Lipids were extracted from plasma by the Folch method and then separated by thin‐layer chromatography on Sorbfil silica gel plates (IMID Ltd, Krasnodar, Russia) in a hexane:diethyl ether:methanol:glacial acetic acid mixture in a ratio of 9:2:0.2:0.3. The lipid fractions were extracted from the silica gel. The quantity of lipids in each fraction was determined based on carbon by combustion with sulfuric acid at 200°C followed by photometry at 405 nm. The percentage ratio of the lipid fractions was calculated.

### 2.7 Enzyme‐linked immunosorbent assay of antibodies against HDL in rabbit serum

Plates were coated overnight at 4°C with human native HDL (Kalen Biomedical) (10 μg/mL). Serum samples were added in serial dilution with PBS, pH 7.2/Tween‐20 and incubated for 3 hours at room temperature. The plates were incubated for 1 hour at room temperature with 100 mL of sheep anti‐rabbit immunoglobulin (Ig) (IgG, IgM, IgA) conjugated to horseradish peroxidase (IMTEC, Russia) diluted 1:8000 in PBS/Tween‐20. Then the substrate mixture was added. At every step, the plates were washed three times with PBS containing Tween‐20. Absorbance was read at 492 nm.

### 2.8 Statistical analysis of the data

The significance of differences was assessed by the Student *t* tests.

## 3 RESULTS

### 3.1 Cartilaginous and adipocytic metaplasia of the aortic wall in rabbits immunized with hnHDL

**Figure 1:**
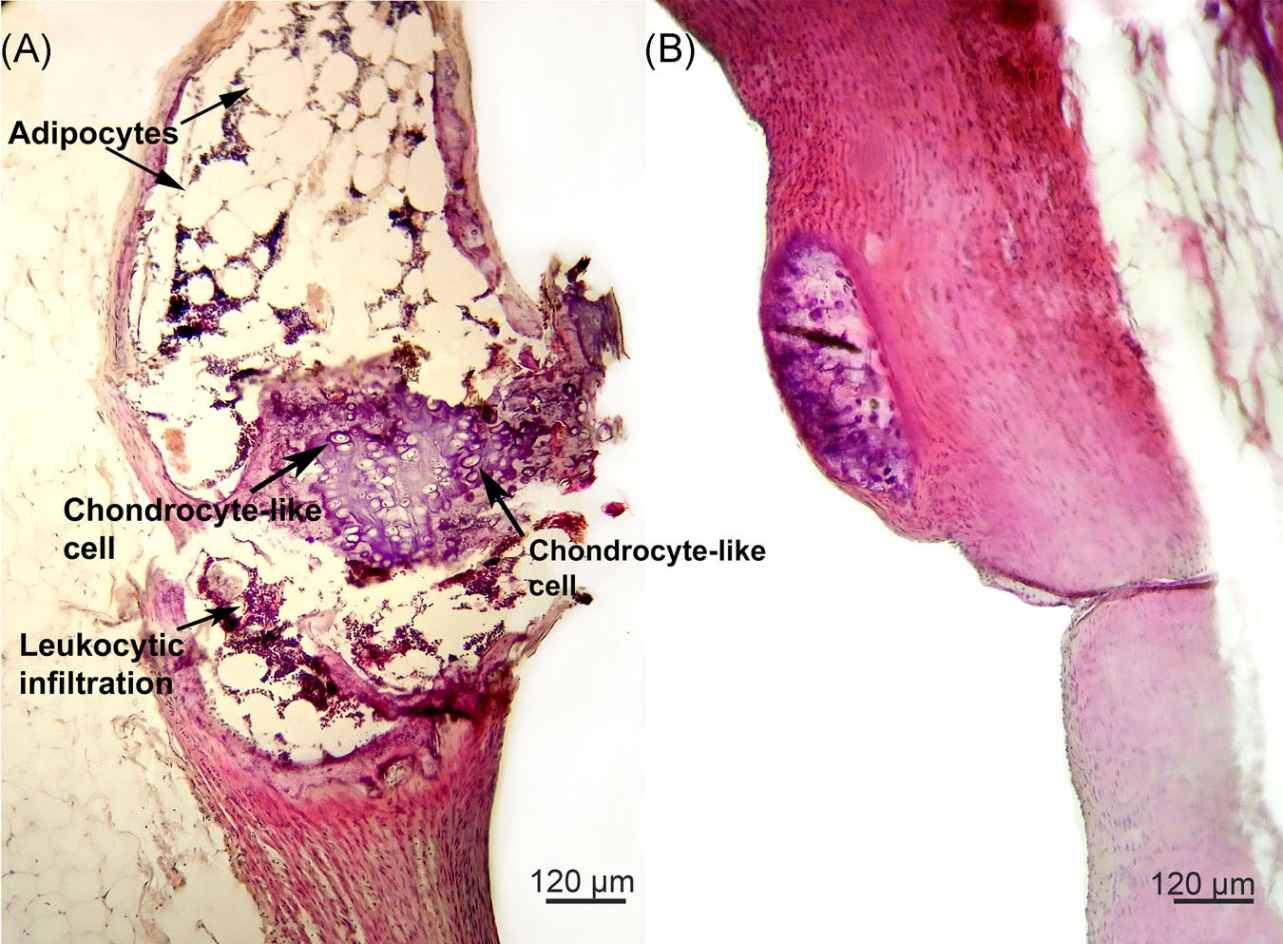
Metaplasia of the aortic wall in rabbits immunized with human native high‐density lipoprotein. A, The entire aortic wall has undergone metaplasia. B, The area of metaplasia is located directly under the intima. Staining with hematoxylin and eosin

**Figure 2:**
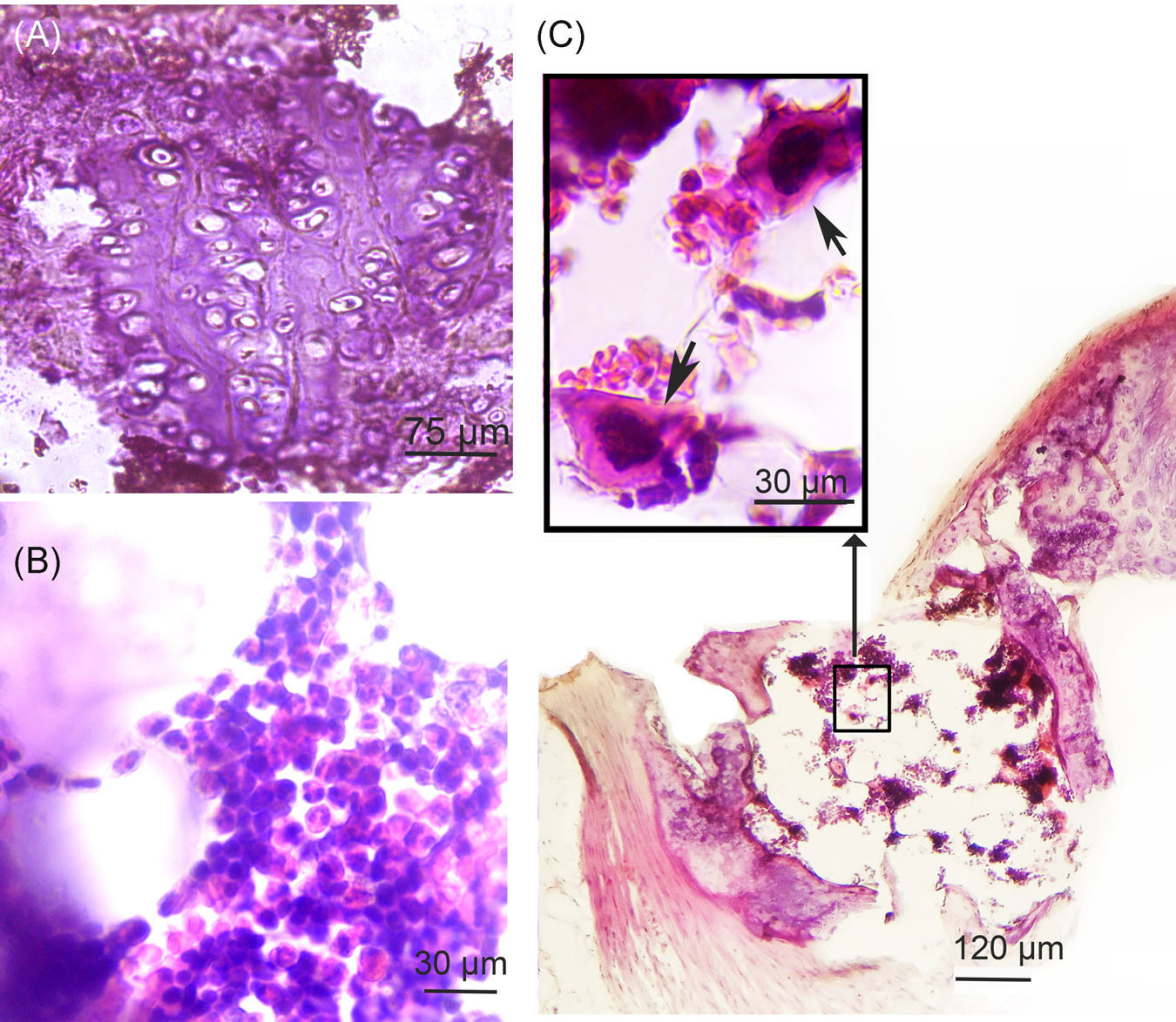
Cell types in the area of aortic metaplasia in human native high‐density lipoprotein‐immunized rabbits. A, Chondrocyte‐like cells in the aortic wall. Enlarged fragments of the aortic wall shown in Figure 1A. B, Adipocytes and leukocytic infiltration. Enlarged fragments of the aortic wall shown in Figure 1A. C, Area of aortic metaplasia in which macrophages (indicated with an arrow) are detected. The majority of adipocytes have been destroyed

Histological analysis of the wall of the aortic arch, the thoracic aorta, and the abdominal aorta in rabbits immunized with hnHDL revealed metaplasia in the wall of the aortic arch (Figure 1). Figure 1A shows the largest change that was found. It is evident that on an approximately 1‐mm section of the aorta, the intima and media are completely replaced by cells that are not typical for a normal vessel wall. The entire thickness of the media in this section of the aorta is taken up with adipocytes (Figure 1A) and chondrocytes (Figures 1A and 2A) and infiltrated with leukocytes (Figures 1A and 2B). It is evident that the metaplasia section of the aortic wall is limited by fibers along the periphery (Figure 1A). Figure 2C shows macrophages in the section of the aorta that has undergone metaplasia.

Figure 1B shows a section of the aorta that contains a formation similar to that presented in Figure 1A but significantly smaller in size. This slice was obtained from another rabbit. It is evident that the formation is located directly under the intima. This finding indicates that the metaplasia of the rabbit aortic wall that develops in response to immunization with hnHDL may start under the intima and then extend throughout the entire thickness of the media. No changes were found in the thoracic or abdominal section of the aorta. The aortic walls of the IFA‐injected or NaCl injected rabbits had no changes.

Atherosclerotic plaques in human blood vessels are known to be a lipid‐rich necrotic core located under the intima. However, the disease mechanisms and histology of plaque development associated with atherosclerosis remain incredibly complex and not entirely understood.^11^ Recent investigations have indicated bone formation within plaques of human coronary vessels.^11^ Brown adipocytes, which pattern heterotopic bone formation, were present within the atherosclerotic lesions.^11^ Human atherosclerotic plaques are infiltrated with leukocytes and may include chondrocytes.^12^, ^13^, ^14^ Consequently, the areas of сartilaginous and adipocytic metaplasia and leukocytic infiltration found in the aortic wall of rabbits immunized with hnHDL are similar in cellular composition to human atherosclerotic plaques.

### 3.2 Proteoglycan deposits in the aortic wall in rabbits immunized with hnHDL

Sections of the aorta from rabbits immunized with hnHDL were stained with pentachrome for the simultaneous staining of collagen and sulfated mucopolysaccharides. This stain colors collagen fibers red, sulfated mucopolysaccharides violet, erythrocytes yellow, muscles orange, and the core green.

**Figure 3:**
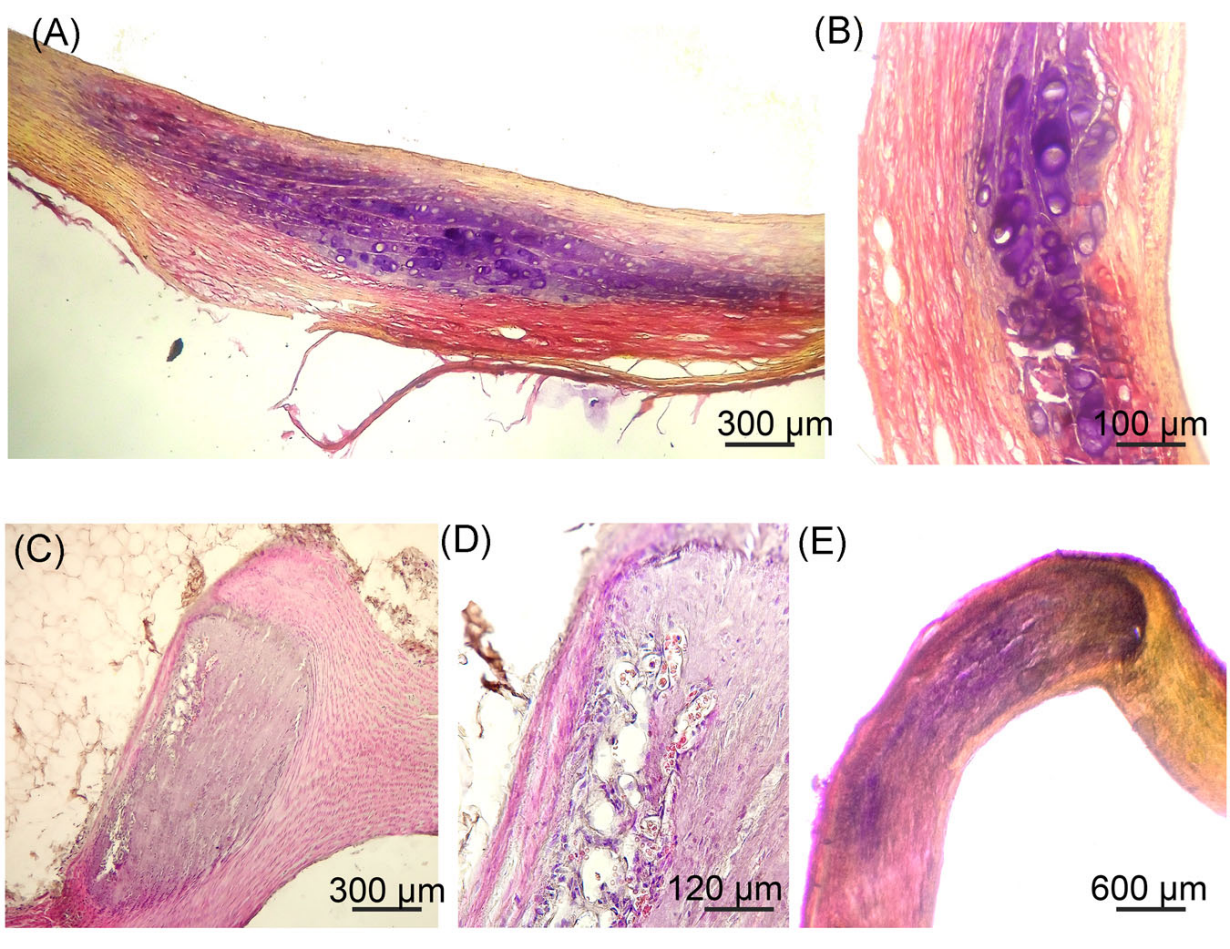
Proteoglycan deposits in the aortic wall of human native high‐density lipoprotein‐immunized rabbits. A, Deposition of proteoglycans (purple staining) is observed around chondrocyte‐like cells. B, Enlarged fragment of the area shown in (A). C, Area of aorta containing a basophilic region and adipocytes but containing no chondrocyte‐like cells. D, Enlarged fragment of the area shown in (C). E, Layer‐by‐layer section of the basophilic region shown in (C), stained for proteoglycans. A, B, E, Pentachrome staining. C, D, Hematoxylin and eosin staining

In the aortic wall of hnHDL‐immunized rabbits, areas were found that contained proteoglycan deposits (violet color) (Figure 3A,B). The violet coloration is localized around chondrocyte‐like cells located in the thickness of the media (Figure 3A,B). It is well known that proteoglycans are secreted by chondrocytes; therefore proteoglycan deposits in the aortic wall are no surprise when there are chondrocytes present.

When the aortic wall of HDL‐immunized rabbits was stained with hematoxylin, extensive basophilic areas were found that contain no chondrocytes (Figure 3C) but have an abnormal wall structure (Figure 3D). When basophilic areas are stained with hematoxylin, they are usually considered proteoglycan deposits in the intercellular space as well as calcifications, since proteoglycans promote calcium deposits.^15^ Pentachrome staining of sequential layer‐by‐layer sections of this basophilic region of the aorta identified a positive response to proteoglycans (Figure 3E). However, no chondrocytes, the source of proteoglycans, were detected in this region. Only some adipocytes were found in this region (Figure 3D). At the same time, it is known that in addition to chondrocyte‐like cells, smooth muscle cells of secretory phenotype can also secrete proteoglycans.^12^ Proceeding from this fact, we can suggest that the smooth muscle cells in this section of the aorta changed phenotype from contractile to secretory. Smooth muscle cells in a proteoglycan‐rich matrix is a progressive atherosclerotic lesion. Extracellular proteoglycans, secreted by smooth muscle cells, bind lipids and progressively increase their lipid‐binding capacity by extension of their disaccharide arms.^16^

In sum, the proteoglycan deposits in the rabbit aortic wall that were found in hnHDL‐immunized rabbits are aortic lesions similar to what occurs in the human atherosclerotic process.

### 3.3 Histological analysis of the myocardium

**Figure 4:**
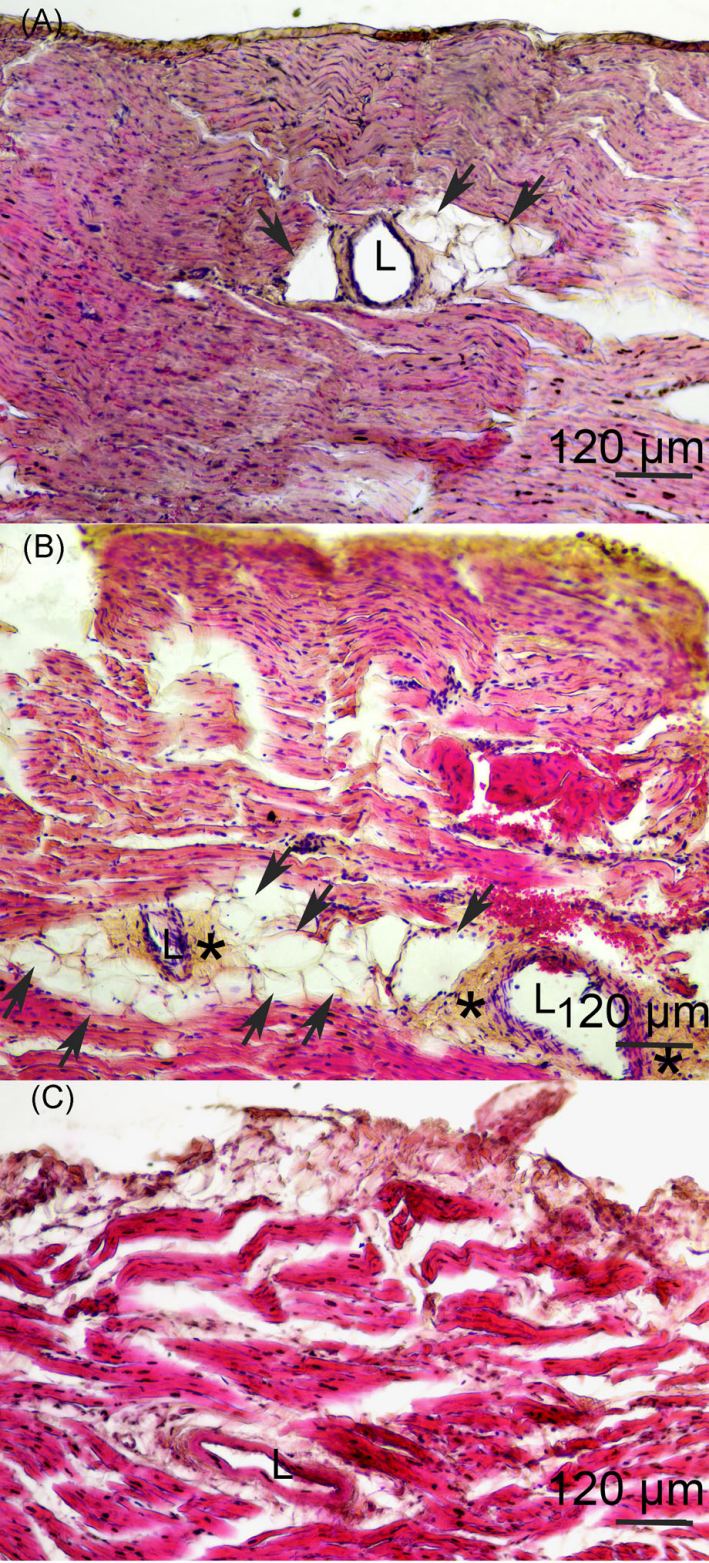
Coronary vessels. A, B, Human native high‐density lipoprotein‐immunized rabbits. C, Incomplete Freund's adjuvant‐injected rabbits staining with hematoxylin and eosin. Adipocytes are indicated with an arrow. *Thickening of the adventitia of the coronary vessel. L, lumen

Figure 4 shows areas of myocardial sections that contain coronary vessels. Hypertrophy and hyperplasia of fatty tissue were detected around the coronary vessels (Figure 4A,B). Furthermore, it is evident that the adventitia in the coronary vessels in hnHDL‐immunized rabbits is a great deal thicker (Figure 4B).

### 3.4 Cholesterol and plasma lipid composition in rabbits immunized with hnHDL

**Figure 5:**
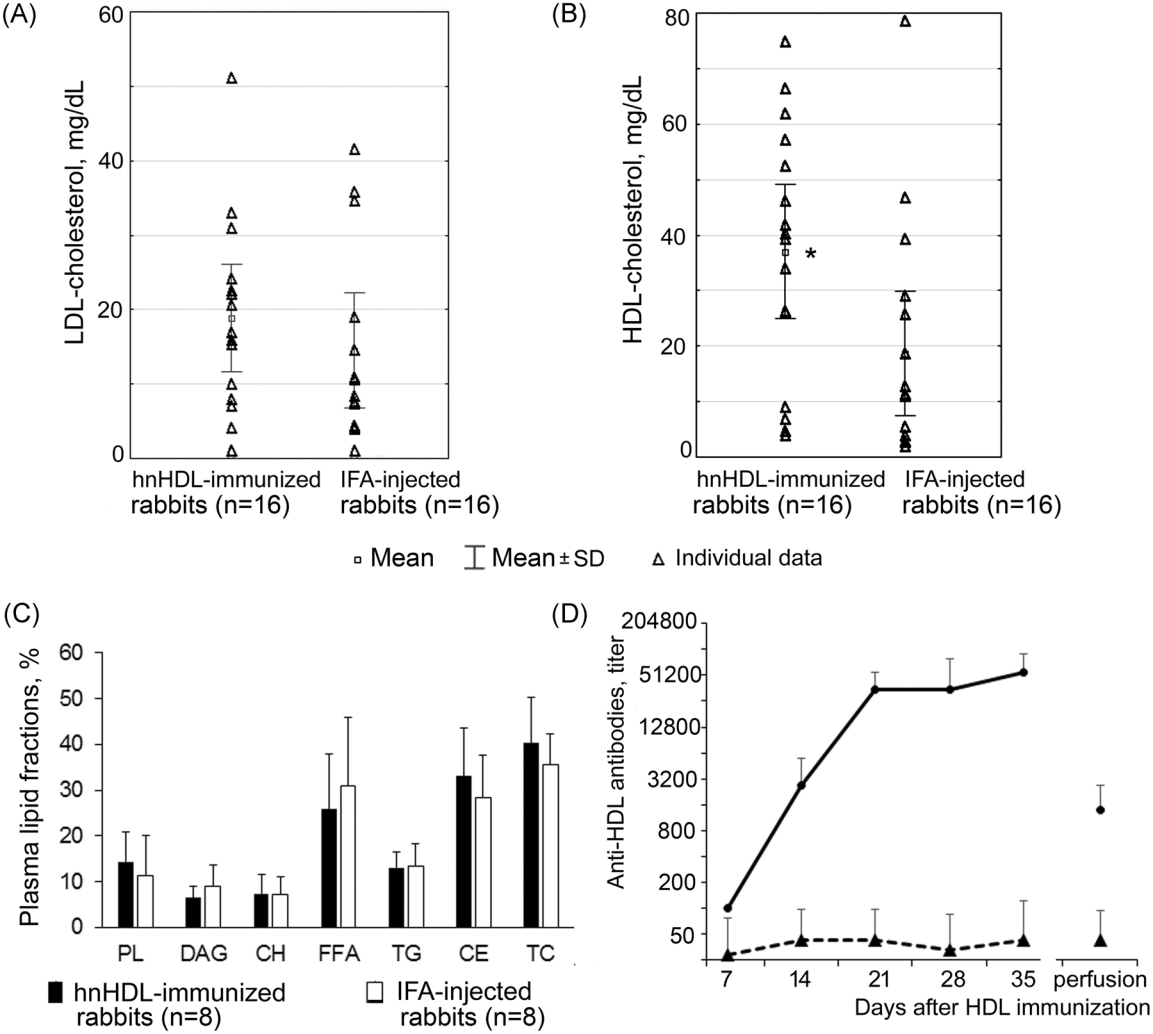
Plasma lipids in hnHDL‐immunized rabbits. A, LDL‐cholesterol levels. The mean of serum samples obtained from four rabbits in each group 2, 4, 6, and 8 weeks after immunization is shown. The data are presented as mean ± SD. B, HDL cholesterol levels. The mean of serum samples obtained from four rabbits in each group 2, 4, 6, and 8 weeks after immunization is shown. The data are presented as mean ± SD. *Statistically significant in relation to IFA‐immunized rabbits, *t*‐test; *P* ≤ .05. C, Ratio of the plasma lipid fractions. The data are presented as mean ± SD. The mean of serum samples obtained from four rabbits in each group 4 and 8 weeks after immunization is shown. The data are presented as mean ± SD. D, Anti‐hnHDL antibodies in the blood of rabbits immunized with hnHDL. The data are presented as mean ± SD. CE, cholesterol esters; CH, cholesterol; DAG, diglycerides; FFA, free fatty acids; hnHDL, human native high‐density lipoprotein; IFA, incomplete Freund's adjuvant; LDL, low‐density lipoprotein; n, number of tested sera; PL, phospholipids; TC, total cholesterol; TG, triglycerides

In rabbits immunized with hnHDL, the LDL‐cholesterol level (Figure 5A) and the HDL‐cholesterol level (Figure 5B) were studied at 2, 4, 6, and 8 weeks post‐HDL immunization. In addition, the ratio of serum lipid fractions was studied in the rabbits at 4 and 8 weeks after immunization (Figure 5C).

There was no difference in serum LDL‐cholesterol levels between the rabbits immunized with hnHDL and those that received an injection of IFA, and these levels were within normal limits for rabbits.^17^ Blood HDL‐cholesterol levels in hnHDL‐immunized rabbits were higher than in the rabbits that had received an injection of IFA (Figure 5B).

In addition, no differences were found in the serum lipid composition of hnHDL‐immunized rabbits versus IFA‐injected rabbits (Figure 5C). Thus, atherosclerosis in hnHDL‐immunized rabbits takes place against a background of normal serum LDL‐cholesterol level.

### 3.5 Antibodies against hnHDL in the blood of immunized rabbits

In the blood of rabbits immunized with hnHDL, antibodies against this antigen were detected (Figure 5D). Antibodies against hnHDL appear in the rabbits’ blood on day 7 after immunization and continue to be detected at the time of perfusion, that is, 39 to 47 weeks after immunization. No antibodies against HDL nor changes in the aortic wall were found in the rabbits receiving the IFA injection.

## 4 DISCUSSION

The purpose of this study was to test whether it is possible to induce atherosclerosis in rabbits with normal plasma cholesterol by immunizing them with native high‐density lipoproteins. Previously, an increase in visceral fat volume, including the appearance of pericardial fat and changes in the aortic wall similar to atherosclerotic ones were able to be induced in rats by immunizing with native human HDL, but no genuine atherosclerotic plaques developed.

Immunization of rabbits with hnHDL was found to cause adipocytic and chondrocytic metaplasia in the rabbit aortic wall, proteoglycan deposits around vascular smooth muscle cells (VSMCs) and chondrocyte‐like cells, and leukocytic infiltration in areas of the lesion. It is known that VSMCs do not undergo terminal differentiation and when certain micro‐environmental stimuli are present, VSMCs can undergo transdifferentiation into chondrocytes and adipocytes, and can also change from the contractile to the synthetic phenotype.^12^, ^14^

Atherosclerotic changes in the aortic wall of hnHDL‐immunized rabbits are found both under the intima and in the media. A number of studies have shown that not only the intima but also the media and the adventitia can be involved in the formation of atherosclerotic lesions.^18^, ^19^ Therefore, the changes in the aortic wall observed in HDL‐immunized rabbits are similar to atherosclerotic changes in the human aortic wall.

We were unable to find any similar studies in the literature showing that the immune response against HDL induces atherosclerosis, except for the fact that in patients the titer of circulating ApoA1‐reactive IgG antibodies is a superior predictor of major cardiac events.^20^, ^21^ Therefore, a rabbit model of atherosclerosis induced by immunization with hnHDL is a new experimental model.

The experimental model of rabbit atherosclerosis we obtained, as well as other experimental models of autoimmune disease induced by immunization with proteins similar to the autoantigen targets, do not themselves reveal the mechanisms of the induction and development of aggressive autoimmune responses that damage tissues. At the same time, these models allow us to understand which antigens are the target of autoimmune attack in a particular disease. While for such diseases as rheumatoid arthritis and multiple sclerosis the target autoantigens have been determined, and collagen‐induced arthritis and experimental autoimmune encephalomyelitis models have confirmed that the target autoantigens of the autoimmune attack are type II collagen and myelin proteins, respectively, the specificity of the immune responses that induce atherosclerosis was not previously clear. The model we obtained characterizes HDL as the target antigen of the immune attack in atherosclerosis, and the immune response against HDL as atherogenic.

The mechanism of the atherogenic effect of anti‐HDL antibodies remains an open question. How the immune response against HDL induces the transdifferentiation of smooth muscle cells into adipocytes, chondrocytes, and synthetic cells, and ultimately the formation of atherosclerotic plaques, is a challenge for future studies.

HDL‐induced model of atherosclerosis raises the question of whether HDL immunization of rabbits corresponds to the mechanism by which atherosclerosis is induced in humans. Since human atherosclerosis develops without a clear inducer, it is possible that HDL immunization of rabbits, thereby triggering an immune response against HDL, imitates the disruption of mechanisms regulating HDL‐specific autoreactive lymphocytes. In turn, questions regarding the mechanisms of regulating autoreactive lymphocytes and the mechanisms of impairing regulation remain key issues in immunology.

The rabbit model of atherosclerosis induced by immunization with hnHDL has been relieved of the deficiencies for which the hypercholesterol model of rabbit atherosclerosis has been criticized. First, rabbit atherosclerosis induced by hnHDL immunization develops without an increase in plasma LDL cholesterol, while the development of diet‐induced atherosclerosis in rabbits is achieved by creating an extremely high plasma LDL‐cholesterol level in the animals that is 10 times normal and is not encountered in human patients with hypercholesterolemia.^1^ Second, in the hnHDL‐immunized rabbits, we were unable to detect foam cells, which form the basis of atherosclerotic lesions in the intima of rabbits on a high‐cholesterol diet but are rare in human atherosclerosis.^1^

Previously, hnHDL‐immunized rats that demonstrated atherosclerosis‐like changes in the aortic wall, like atherosclerotic hnHDL‐immunized rabbits, did not exhibit elevated plasma cholesterol levels. The fact that experimental atherosclerosis can be induced by immunization with lipoproteins without an increase in plasma LDL‐cholesterol level allows us to hypothesize that excess plasma LDL cholesterol is neither the sole nor the principal cause of atherogenesis. The rabbit and rat atherosclerosis models that we obtained support the criticism of the cholesterol hypothesis of atherosclerosis that has been cogently presented in the works of Ravnskov et al^22^, ^23^, ^24^ From their analysis of numerous studies, Ravnskov et al^22^ concluded that people with low LDL‐cholesterol levels become just as atherosclerotic as people with high LDL‐cholesterol levels and their risk of suffering from cardiovascular disease is the same or higher.

At the same time, we found that hnHDL‐immunized rabbits have blood HDL‐cholesterol levels about twice those of the IFA‐injected rabbits. The increase in HDL cholesterol is comparable to that observed in rabbits fed a high‐cholesterol diet.^17^ The cause of the increase in blood HDL cholesterol in hnHDL‐immunized rabbits may be a compensatory response to the removal from the blood of HDL opsonized with anti‐HDL antibodies present in the blood of immunized rabbits.

## 5 CONCLUSION

Immunization with human native HDL induces atherosclerosis‐like lesions in the rabbit aorta such as adipocytic and chondrocytic metaplasia, proteoglycan deposits, and leukocytic infiltration. Atherosclerosis‐like lesions develop in the aorta of hnHDL‐immunized rabbits against a background of normal blood LDL‐cholesterol level. Consequently, the immune response against HDL may be an independent cause of atherogenesis, and HDL is the potential target of the immune attack that leads to atherosclerosis. A rabbit model of atherosclerosis induced by immunization with hnHDL can be widely used to examine the mechanisms occurring during atherogenesis.

## CONFLICT OF INTERESTS

The authors declare that there are no conflict of interests.

## AUTHOR CONTRIBUTIONS

KF: investigation, data curation, visualization, and writing. LB: methodology, writing (review and editing), visualization, validation, and supervision. IM: conceptualization and writing. AТ and AS: investigation and visualization. AZ, TK, NA, and AG: investigation. All authors read and approved the manuscript.

## Data Availability

The data that support the findings of this study are available from the corresponding author upon reasonable request.
